# Supplementary material for: Designing conservation strategies to preserve the genetic diversity of Astragalus edulis Bunge, an endangered species from western Mediterranean region
Source: PeerJ. 2016 Jan 18;4:e1474. doi: 10.7717/peerj.1474 (PMC4736990; doi:10.7717/peerj.1474)
Supplement: Supplemental Information 3 — Appendix 1. Probabilities of loss of rare alleles when all populations of Astragalus edulis are considered as one single management unit and preferred sampling area. [file peerj-04-1474-s003.pdf]

Appendix 1. Probabilities of loss of 273 rare alleles when all populations of *Astragalus edulis* are considered as one single management unit and preferred sampling area. N, number of populations where each allele was found; A_N allele number; Geographical groups: IP=Iberian Peninsula; M=Morocco; CI=Canary Islands; L_o, observed probability of loss of the allele; L_e, expected probability of loss of the allele; PSA, preferred sampling area.

A_N	N	IP	M	CI	Lo	Le	PSA
B3	3	1	2	0	0.95103016	0.98346744	IP
B8	3	1	0	2	0.93515798	0.97801577	IP
B12	1	1	0	0	0.98891975	0.98894859	IP
B13	3	2	1	0	0.95103016	0.98346744	IP
B16	3	3	0	0	0.95103016	0.98346744	IP
B18	3	0	3	0	0.93515798	0.97801577	M
B21	2	1	1	0	0.97796228	0.98894859	IP
B31	3	1	2	0	0.73509189	0.9047041	IP
B33	1	0	1	0	0.99445216	0.9944594	M
B37	2	1	1	0	0.97796228	0.98894859	IP
B40	1	0	0	1	0.98891975	0.98894859	CI
B44	2	0	1	1	0.96708102	0.98346744	M
B46	1	1	0	0	0.99445216	0.9944594	IP
B47	1	0	0	1	0.99445216	0.9944594	CI
B53	1	0	0	1	0.98891975	0.98894859	CI
B55	3	2	1	0	0.91950716	0.97259343	IP
B56	1	0	0	1	0.99445216	0.9944594	CI
B58	1	0	1	0	0.99445216	0.9944594	M
B66	2	1	1	0	0.97796228	0.98894859	IP
B67	3	0	0	3	0.90407522	0.96720029	CI
B68	1	0	1	0	0.99445216	0.9944594	M
B70	1	0	1	0	0.99445216	0.9944594	M
B72	3	0	3	0	0.95103016	0.98346744	M
B73	2	0	0	2	0.95629082	0.97801577	CI

B74	2	0	0	2	0.97796228	0.98894859	CI
B81	1	0	0	1	0.98891975	0.98894859	CI
B82	1	0	1	0	0.99445216	0.9944594	M
B83	1	0	0	1	0.99445216	0.9944594	CI
B85	1	0	1	0	0.99445216	0.9944594	M
B87	1	0	0	1	0.99445216	0.9944594	CI
B88	3	1	2	0	0.95103016	0.98346744	IP
B89	2	2	0	0	0.97796228	0.98894859	IP
B90	3	0	0	3	0.95103016	0.98346744	CI
B91	1	1	0	0	0.99445216	0.9944594	IP
B93	1	1	0	0	0.98891975	0.98894859	IP
B94	1	0	0	1	0.99445216	0.9944594	CI
B99	3	1	1	1	0.93515798	0.97801577	IP
B100	1	0	0	1	0.99445216	0.9944594	CI
B105	2	0	2	0	0.97796228	0.98894859	M
B107	1	1	0	0	0.99445216	0.9944594	IP
B108	1	0	0	1	0.99445216	0.9944594	CI
B109	2	1	1	0	0.96708102	0.98346744	IP
B110	1	1	0	0	0.99445216	0.9944594	IP
B111	2	2	0	0	0.97796228	0.98894859	IP
B115	1	1	0	0	0.99445216	0.9944594	IP
B119	2	2	0	0	0.97796228	0.98894859	IP
B120	1	0	0	1	0.99445216	0.9944594	CI
B125	3	0	3	0	0.84448757	0.94591649	M
B129	3	0	3	0	0.93515798	0.97801577	M
B130	1	0	0	1	0.98891975	0.98894859	CI
B136	3	1	2	0	0.91950716	0.97259343	IP
B139	2	0	1	1	0.96708102	0.98346744	M
B142	3	0	3	0	0.93515798	0.97801577	M
B146	1	0	1	0	0.99445216	0.9944594	M
B147	2	0	0	2	0.97796228	0.98894859	CI
B150	3	1	2	0	0.91950716	0.97259343	IP

B152	2	1	1	0	0.97796228	0.98894859	IP
B157	1	0	1	0	0.99445216	0.9944594	M
B162	2	1	1	0	0.97796228	0.98894859	IP
B166	2	0	2	0	0.97796228	0.98894859	M
B169	1	0	1	0	0.98891975	0.98894859	M
B171	3	1	2	0	0.76126463	0.91484074	IP
B173	3	1	1	1	0.93515798	0.97801577	IP
B174	2	1	1	0	0.97796228	0.98894859	IP
B175	3	2	1	0	0.95103016	0.98346744	IP
B176	1	1	0	0	0.99445216	0.9944594	IP
B178	1	0	0	1	0.99445216	0.9944594	CI
B179	1	0	1	0	0.99445216	0.9944594	M
B186	2	2	0	0	0.97796228	0.98894859	IP
B187	2	0	1	1	0.96708102	0.98346744	M
B189	3	1	2	0	0.93515798	0.97801577	IP
B190	3	1	2	0	0.95103016	0.98346744	IP
B192	3	1	2	0	0.91950716	0.97259343	IP
B193	2	0	2	0	0.96708102	0.98346744	M
B194	1	1	0	0	0.94521605	0.94591649	IP
B196	2	1	1	0	0.97796228	0.98894859	IP
B197	1	1	0	0	0.98891975	0.98894859	IP
B198	1	1	0	0	0.99445216	0.9944594	IP
B199	1	0	1	0	0.98891975	0.98894859	M
B200	2	0	2	0	0.97796228	0.98894859	M
B201	3	0	3	0	0.95103016	0.98346744	M
B202	1	0	1	0	0.99445216	0.9944594	M
B203	2	0	2	0	0.96708102	0.98346744	M
B205	2	0	2	0	0.97796228	0.98894859	M
B207	1	0	0	1	0.99445216	0.9944594	CI
B208	1	0	1	0	0.99445216	0.9944594	M
B209	3	1	1	1	0.93515798	0.97801577	IP
B210	1	0	1	0	0.9614892	0.96183617	M

B211	1	0	1	0	0.99445216	0.9944594	M
B212	3	3	0	0	0.78820936	0.92508757	IP
B213	3	3	0	0	0.67292149	0.87983711	IP
B214	1	0	0	1	0.99445216	0.9944594	CI
B215	1	0	1	0	0.99445216	0.9944594	M
B217	2	0	2	0	0.97796228	0.98894859	M
B220	1	0	1	0	0.98891975	0.98894859	M
B221	2	0	2	0	0.88326639	0.94066698	M
B222	3	0	0	3	0.67292149	0.87983711	CI
B223	2	0	2	0	0.97796228	0.98894859	M
B224	1	0	1	0	0.99445216	0.9944594	M
B225	3	0	2	1	0.90407522	0.96720029	M
B227	1	0	0	1	0.99445216	0.9944594	CI
B228	1	0	1	0	0.99445216	0.9944594	M
B229	1	0	0	1	0.99445216	0.9944594	CI
B230	2	0	2	0	0.88326639	0.94066698	M
B231	1	0	0	1	0.99445216	0.9944594	CI
B232	2	0	2	0	0.84346366	0.91995031	M
B234	3	0	3	0	0.60416138	0.8508716	M
B236	1	0	1	0	0.98891975	0.98894859	M
B238	1	0	1	0	0.99445216	0.9944594	M
B239	1	1	0	0	0.92373457	0.92508757	IP
B240	2	2	0	0	0.84346366	0.91995031	IP
B243	1	0	0	1	0.99445216	0.9944594	CI
B245	1	0	0	1	0.99445216	0.9944594	CI
B251	3	0	0	3	0.90407522	0.96720029	CI
B252	2	0	0	2	0.97796228	0.98894859	CI
G5	2	1	1	0	0.97796228	0.98894859	IP
G15	3	2	1	0	0.93515798	0.97801577	IP
G17	2	2	0	0	0.95629082	0.97801577	IP
G22	2	0	2	0	0.96708102	0.98346744	M
G24	1	0	0	1	0.99445216	0.9944594	CI

G38	1	1	0	0	0.99445216	0.9944594	IP
G41	1	0	1	0	0.99445216	0.9944594	M
G46	1	0	1	0	0.98891975	0.98894859	M
G53	2	1	1	0	0.80502156	0.89967675	IP
G54	3	2	1	0	0.87385822	0.95650093	IP
G55	1	0	1	0	0.95604938	0.95650093	M
G59	1	0	0	1	0.99445216	0.9944594	CI
G65	1	0	1	0	0.98891975	0.98894859	M
G67	2	0	2	0	0.96708102	0.98346744	M
G69	3	0	0	3	0.73509189	0.9047041	CI
G70	2	0	2	0	0.97796228	0.98894859	M
G71	3	0	3	0	0.70967335	0.89467652	M
G72	2	0	1	1	0.95629082	0.97801577	M
G73	3	2	0	1	0.88885972	0.96183617	IP
G74	1	1	0	0	0.99445216	0.9944594	IP
G76	2	0	2	0	0.74984107	0.87007719	M
G77	3	2	1	0	0.87385822	0.95650093	IP
G78	1	0	0	1	0.99445216	0.9944594	CI
G79	1	1	0	0	0.99445216	0.9944594	IP
G80	2	1	1	0	0.97796228	0.98894859	IP
G84	3	0	2	1	0.87385822	0.95650093	M
G87	2	1	1	0	0.97796228	0.98894859	IP
G91	3	0	3	0	0.91950716	0.97259343	M
G92	2	1	1	0	0.97796228	0.98894859	IP
G93	2	0	2	0	0.96708102	0.98346744	M
G94	3	0	3	0	0.84448757	0.94591649	M
G95	3	1	2	0	0.93515798	0.97801577	IP
G108	2	0	2	0	0.97796228	0.98894859	M
G115	1	0	0	1	0.99445216	0.9944594	CI
G116	2	0	2	0	0.97796228	0.98894859	M
G117	2	2	0	0	0.97796228	0.98894859	IP
G127	1	0	1	0	0.99445216	0.9944594	M

G128	3	0	1	2	0.93515798	0.97801577	M
G129	3	0	2	1	0.93515798	0.97801577	M
G130	3	0	3	0	0.69724143	0.88970326	M
G135	1	0	1	0	0.99445216	0.9944594	M
G137	1	0	0	1	0.99445216	0.9944594	CI
G138	1	0	1	0	0.99445216	0.9944594	M
G141	1	0	0	1	0.98891975	0.98894859	CI
G143	1	1	0	0	0.99445216	0.9944594	IP
G144	3	2	1	0	0.91950716	0.97259343	IP
G147	1	0	0	1	0.98891975	0.98894859	CI
G153	1	0	1	0	0.97790123	0.97801577	M
G154	2	0	1	1	0.97796228	0.98894859	M
G155	1	0	0	1	0.99445216	0.9944594	CI
G157	3	0	3	0	0.76126463	0.91484074	M
G159	3	0	2	1	0.88885972	0.96183617	M
G160	1	0	1	0	0.98891975	0.98894859	M
G163	1	0	1	0	0.98891975	0.98894859	M
G164	3	0	1	2	0.90407522	0.96720029	M
G165	3	1	1	1	0.95103016	0.98346744	IP
G175	2	1	1	0	0.97796228	0.98894859	IP
G176	1	0	1	0	0.99445216	0.9944594	M
G177	2	0	0	2	0.97796228	0.98894859	CI
G180	1	0	0	1	0.9614892	0.96183617	CI
G184	3	0	2	1	0.95103016	0.98346744	M
G185	1	0	0	1	0.98891975	0.98894859	CI
G187	1	0	0	1	0.99445216	0.9944594	CI
G188	1	1	0	0	0.99445216	0.9944594	IP
G191	1	1	0	0	0.99445216	0.9944594	IP
G192	1	1	0	0	0.99445216	0.9944594	IP
G193	1	0	0	1	0.98891975	0.98894859	CI
G198	1	1	0	0	0.99445216	0.9944594	IP
G199	2	0	2	0	0.96708102	0.98346744	M

G212	3	2	0	1	0.95103016	0.98346744	IP
G214	3	3	0	0	0.93515798	0.97801577	IP
G217	1	1	0	0	0.99445216	0.9944594	IP
G218	1	1	0	0	0.97790123	0.97801577	IP
G219	1	1	0	0	0.99445216	0.9944594	IP
G220	1	0	0	1	0.98891975	0.98894859	CI
G221	1	1	0	0	0.99445216	0.9944594	IP
G222	1	0	0	1	0.99445216	0.9944594	CI
G223	1	1	0	0	0.99445216	0.9944594	IP
G224	1	0	0	1	0.99445216	0.9944594	CI
G225	1	1	0	0	0.99445216	0.9944594	IP
G226	1	0	1	0	0.98891975	0.98894859	M
G227	1	0	0	1	0.99445216	0.9944594	CI
G228	3	1	2	0	0.95103016	0.98346744	IP
G231	1	0	1	0	0.99445216	0.9944594	M
G233	2	0	2	0	0.95629082	0.97801577	M
G235	2	0	0	2	0.94559117	0.97259343	CI
G236	2	0	2	0	0.96708102	0.98346744	M
G237	2	0	0	2	0.97796228	0.98894859	CI
G240	1	0	1	0	0.98891975	0.98894859	M
G241	1	0	1	0	0.98891975	0.98894859	M
G244	2	0	2	0	0.87318642	0.93544576	M
G245	1	0	1	0	0.99445216	0.9944594	M
G247	1	0	1	0	0.99445216	0.9944594	M
G250	1	0	0	1	0.99445216	0.9944594	CI
G252	2	0	1	1	0.96708102	0.98346744	M
G254	1	0	1	0	0.98891975	0.98894859	M
G255	1	0	0	1	0.99445216	0.9944594	CI
G256	2	0	2	0	0.92446148	0.96183617	M
G259	2	1	1	0	0.97796228	0.98894859	IP
G262	1	0	0	1	0.99445216	0.9944594	CI
G263	1	0	0	1	0.99445216	0.9944594	CI

G266	1	0	0	1	0.99445216	0.9944594	CI
G269	1	0	0	1	0.99445216	0.9944594	CI
G271	1	0	1	0	0.99445216	0.9944594	M
G272	1	0	1	0	0.98891975	0.98894859	M
Y19	2	1	1	0	0.97796228	0.98894859	M
Y110	1	0	1	0	0.98891975	0.98894859	M
Y143	1	1	0	0	0.99445216	0.9944594	IP
Y217	3	3	0	0	0.95103016	0.98346744	IP
Y221	3	3	0	0	0.91950716	0.97259343	IP
Y246	3	0	0	3	0.93515798	0.97801577	CI
Y275	2	0	0	2	0.97796228	0.98894859	CI
Y302	3	3	0	0	0.93515798	0.97801577	IP
Y314	3	1	2	0	0.95103016	0.98346744	IP
Y328	2	1	1	0	0.96708102	0.98346744	IP
Y349	2	2	0	0	0.94559117	0.97259343	IP
Y362	3	3	0	0	0.93515798	0.97801577	IP
Y368	2	2	0	0	0.94559117	0.97259343	IP
Y372	3	2	1	0	0.95103016	0.98346744	IP
Y390	3	1	2	0	0.91950716	0.97259343	IP
Y391	3	3	0	0	0.93515798	0.97801577	IP
Y392	1	1	0	0	0.99445216	0.9944594	IP
Y400	3	3	0	0	0.93515798	0.97801577	IP
Y404	3	1	0	2	0.93515798	0.97801577	IP
Y409	1	1	0	0	0.98340278	0.98346744	IP
Y419	3	3	0	0	0.91950716	0.97259343	IP
Y425	1	0	0	1	0.97790123	0.97801577	CI
Y431	3	2	1	0	0.93515798	0.97801577	IP
Y441	3	3	0	0	0.95103016	0.98346744	IP
Y444	2	2	0	0	0.95629082	0.97801577	IP
Y453	3	2	1	0	0.93515798	0.97801577	IP
Y455	3	3	0	0	0.88885972	0.96183617	IP
Y458	3	3	0	0	0.95103016	0.98346744	IP

Y465	2	2	0	0	0.96708102	0.98346744	IP
Y466	3	3	0	0	0.95103016	0.98346744	IP
Y467	3	2	1	0	0.93515798	0.97801577	IP
Y477	3	2	1	0	0.90407522	0.96720029	IP
Y495	1	1	0	0	0.99445216	0.9944594	IP
Y496	1	0	1	0	0.99445216	0.9944594	M
Y507	2	2	0	0	0.96708102	0.98346744	IP
Y517	3	2	1	0	0.93515798	0.97801577	IP
Y518	3	2	1	0	0.93515798	0.97801577	IP
Y527	3	3	0	0	0.93515798	0.97801577	IP
Y531	3	2	0	1	0.90407522	0.96720029	IP
Y537	2	2	0	0	0.94559117	0.97259343	IP
Y539	2	2	0	0	0.97796228	0.98894859	IP
Y542	3	1	1	1	0.93515798	0.97801577	IP
Y545	3	2	1	0	0.93515798	0.97801577	IP
Y547	3	2	1	0	0.93515798	0.97801577	IP
Y551	2	2	0	0	0.97796228	0.98894859	IP
Y562	3	2	0	1	0.95103016	0.98346744	IP
Y568	3	3	0	0	0.95103016	0.98346744	IP
Y579	3	2	1	0	0.90407522	0.96720029	IP
Y592	2	2	0	0	0.92446148	0.96183617	IP
Y601	3	3	0	0	0.90407522	0.96720029	IP
Y603	3	2	0	1	0.95103016	0.98346744	IP
Y617	2	2	0	0	0.95629082	0.97801577	IP
Y619	3	3	0	0	0.91950716	0.97259343	IP
